# Establishment and Validation of an Efficient *Agrobacterium Tumefaciens*-Mediated Transient Transformation System for *Salix Psammophila*

**DOI:** 10.3390/ijms252312934

**Published:** 2024-12-01

**Authors:** Yanfei Yang, Zhicheng Chen, Jinna Zhao, Guangshun Zheng, Fei Wang, Shaofeng Li, Xingrong Ren, Jianbo Li

**Affiliations:** 1 China National Permanent Scientific Research Base for Warm Temperate Zone Forestry of Jiulong Mountain, Experimental Centre of Forestry in North China, Chinese Academy of Forestry, Beijing 102300, China; 13759225665@163.com (Y.Y.); guangshunzheng@163.com (G.Z.); wangfei@caf.ac.cn (F.W.); lisf@caf.ac.cn (S.L.); 2 College of Forestry, Shanxi Agricultural University, Taigu 030801, China; jinnazz@163.com (J.Z.); 18064256020@163.com (X.R.); 3 Key Laboratory of Forest Ecology and Environment of National Forestry and Grassland Administration, Ecology and Nature Conservation Institute, Chinese Academy of Forestry, Beijing 100091, China; zcchen@caf.ac.cn

**Keywords:** *Salix psammophila*, transient transformation, GUS staining, *SpPP2C80*

## Abstract

*Salix psammophila*, C. Wang & Chang Y. Yang, a desert-adapted shrub, is recognized for its exceptional drought tolerance and plays a vital role in ecosystem maintenance. However, research on *S. psammophila* has been limited due to the lack of an efficient and reliable genetic transformation method, including gene functional studies. The *Agrobacterium*-mediated transient overexpression assay is a rapid and powerful tool for analyzing gene function in plant vivo. In this study, tissue culture seedlings of *S. psammophila* were utilized as the recipient materials, and the plant expression vector pCAMBIA1301, containing the *GUS* reporter gene, was transferred into the seedlings via an *Agrobacterium*-mediated method. To enhance the efficiency of the system, the effects of secondary culture time, *Agrobacterium* concentration, infection time, and co-culture duration on the transient transformation efficiency of *S. psammophila* were explored. The optimal combination for the instantaneous transformation of *S. psammophila* tissue culture seedlings mediated by *Agrobacterium* was determined as follows: a secondary culture time of 30 d, a value of OD_600_ of 0.8, an infection time of 3 h, and a co-culture duration of 48 h. Subsequently, the effectiveness of the transformation system was validated using the *S. psammophila* drought response gene *SpPP2C80*. To further confirm the accuracy of the system, *SpPP2C80*-overexpressing *Arabidopsis* was constructed and drought resistance analysis was performed. The results were consistent with the transient overexpression of *SpPP2C80* in *S. psammophila* tissue culture seedlings, indicating that this system can be effectively employed for studying gene function in *S. psammophila*. These findings provide essential information for investigating gene function in non-model plants and pave the way for advancements in molecular biology research in *S. psammophila*.

## 1. Introduction

*Salix psammophila*, C. Wang & Chang Y. Yang, a shrub species commonly utilized for windbreaks and sand fixation, is widely distributed in arid and semi-arid desert regions of Northern China, including Inner Mongolia, Shaanxi Province, Ningxia Province, and others [1]. In terms of economy, *S. psammophila* is valuable in papermaking and the production of microcrystalline cellulose composite materials. Additionally, as a renewable resource, *S. psammophila* can also be used for power generation [1]. *S. sammophila* has the characteristics of resistance to wind erosion and sand burial and can survive in a desert water shortage environment, with strong stress resistance [2]. Due to its high stress resistance, *S. psammophila* serves as an ideal material for studying plant stress resistance mechanisms [1]. Therefore, it is important to study the functions of stress resistance-related genes in *S. psammophila*.

The *Agrobacterium*-mediated transient transformation of plants is a technology that enables the rapid and high-level expression of target genes [3], extensively applied for gene functional verification [4]. Unlike stable transformation, this method does not rely on the chromosomal integration of heterologous DNA [5], making it simpler, faster, and cost-effective [6]. This technology serves as a powerful tool for investigating plant gene functions without the conventional need for genetic transformation methods [7]. The methods for plant transient transformation systems for transforming exogenous genes are similar to those for stable genetic transformation, including the PEG method, gene gun method, plant virus vector-mediated method, and *Agrobacterium*-mediated method [8]. Compared with other transformation methods, *Agrobacterium*-mediated transient transformation technology stands out for its convenience, batch conversion, low cost, and high effectiveness [9]. The principle is to transform the vector containing the target gene into *Agrobacterium*, and then introduce the infection buffer prepared by *Agrobacterium* into plant cells through various methods to achieve transient expression [8]. After entering *Agrobacterium*, the infection buffer prepared using *Agrobacterium* will be introduced into plant cells through various methods and transiently expressed, with the entire process taking only a few days [10]. *Agrobacterium*-mediated transient transformation method can utilize various explants for transformation. Besides plant leaves, petioles, petals, stem tips, stem segments, callus tissue, and hypocotyl nodes, segments and somatic embryos can be used as explants, and currently, woody plants such as apples, citrus, pears, and peaches can also undergo transient genetic transformation using fruit pulp [11,12]. Furthermore, mulberry trees, Xinjiang wild apples, birch trees, poplar trees, tamarisk trees, cork trees, and oak trees can be soaked in intact plants for instantaneous transformation [5,13]. At present, this technology has been successfully applied to verify the functions of many plant genes, such as *Betula platyphylla* [14], *Populus euphratica* [15], and *Gossypium hirsutum* [16]. And the transient transformation system can also be applied in promoter and transcription factor analysis, as well as protein subcellular localization and interaction [17].

Research has suggested that the efficiency of plants’ transient transformation is influenced by the growth of the transformed recipient material [18], *Agrobacterium* density [16], infection time [19], co-culture duration [20], and other factors [17,18]. In the transformation process, plant genotype, receptor material type, and physiological status are closely related to instantaneous transformation efficiency [8]. *Agrobacterium* serves as a medium for instantaneous transformation, and the concentration of the *Agrobacterium* directly affects the efficiency of instantaneous transformation. The appropriate concentration is conducive to transformation [21], while an excessive concentration of bacterial solution has a certain toxic effect on plants, causing them to soften and wilt, thereby reducing the instantaneous expression level of exogenous genes. The infection time is also a critical factor affecting the transient transformation efficiency; thus, an appropriate infection time is very important [22]. If the infection time of *Agrobacterium* is too long, there will be a substantial amount of *Agrobacterium* residue on the surface of the plant, which may proliferate in the later cultivation process and be difficult to control, leading to plant death. Conversely, if the infection time is too short, the contact between the bacterial solution and the plant will be not sufficient, which leads to the emergence of a significant quantity of non-resistance seedlings [19]. The process of expressing exogenous genes carried by *Agrobacterium* in plant cells is called co-culture. If the co-culture duration is too short, it will lead to the low expression level of exogenous genes, if the co-culture duration is too long, the expressed products will degrade, and neither situations can achieve ideal conversion rates [23].

Plants encounter a variety of abiotic and biotic stresses during their growth and development. Meanwhile, plants have evolved complex regulatory mechanisms to cope with these challenges [24]. The post-translational modification of proteins is one of the most important regulatory strategies, including protein kinase-mediated phosphorylation and protein phosphatase-mediated dephosphorylation [25]. In our previous study, the global transcriptome profiles of *S. psammophila* under drought conditions revealed that *SpPP2C80* was a key drought-responsive gene, but its biological function has not been thoroughly dissected, especially in response to abiotic environmental stimuli [26]. Type 2C protein phosphatases (PP2Cs) are the main protein phosphatases in plants, playing a significant role in regulating abiotic stresses such as drought and cold [27,28]. Under low temperature stress, PP2Cs promote the activity of OST1 (OPEN STOMATA 1) to endow plants with low temperature tolerance [29]. PP2Cs are involved in the regulation of nitrogen and potassium uptake during nutrient deficiency stress [30,31]. In addition, PP2Cs are also involved in the plant resistance of pests and pathogens [32]. It is important to search the molecular mechanism of PP2Cs in the regulation of stress responses for improving plant stress tolerance. Although some PP2Cs are known to be involved in abiotic stresses in plants, and they have been studied in plants such as corn, rice, and apples [28], their function has not been reported in *S. psammophila.*

Currently, the stable inheritance of target genes into plants to obtain transgenic plants is a prevalent method for studying gene function [17]. However, the stable genetic transformation system of *S. psammophila* is not yet perfect, and there are challenges associated with *S. psammophila* transformation. Currently, research on the gene function of *S. psammophila* predominantly relies on model plants such as *Arabidopsis* and poplar [33,34]. Therefore, establishing an efficient transient transformation system mediated by *Agrobacterium* is essential for investigating the stress resistance gene function of *S. psammophila*. In this study, we screened each factor mentioned above and established a high-efficiency transient transformation system of *S. psammophila* mediated by *Agrobacterium*. In addition, based on the overexpression of *S. psammophila* stress resistance gene *SpPP2C80* in the above system, the feasibility of the transient transformation system was preliminarily verified. And the accuracy of the system was verified by the overexpression of *Arabidopsis*. The efficient and rapid transient genetic transformation system of *S. psammophila* can realize the in situ verification of important gene functions in a short time and accelerate the screening and utilization process of special genes of *S. psammophila*.

## 2. Result

### 2.1. Effect of Secondary Culture Time on Transient Transformation Efficiency of S. psammophila

**Figure 1:**
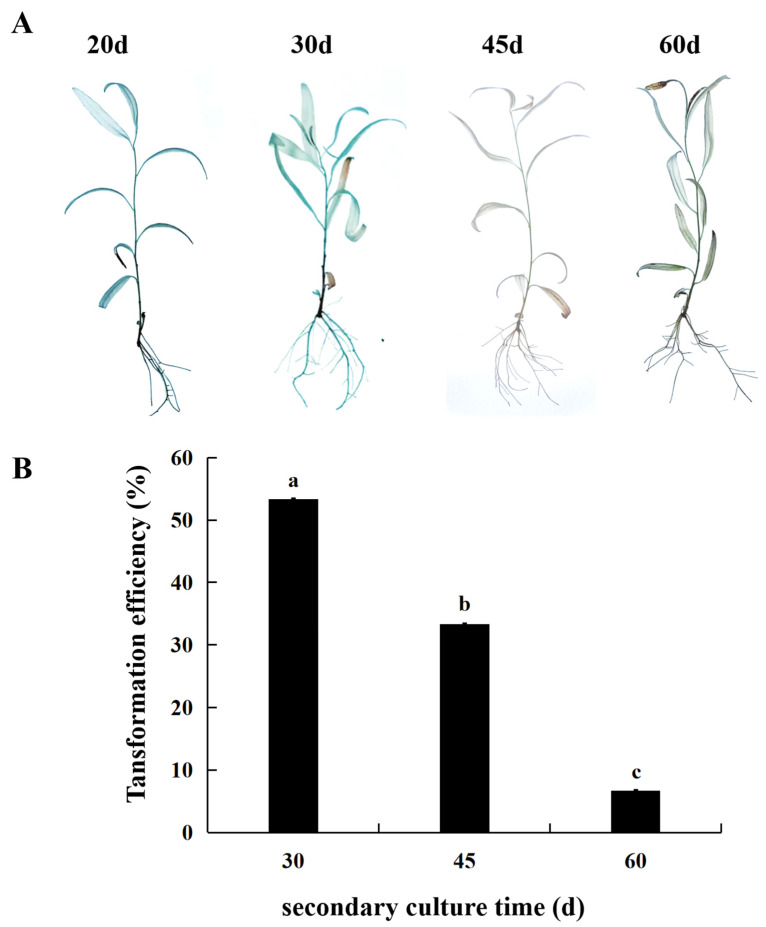
Effect of secondary culture time on transient transformation of *S. psammophila*. (**A**) GUS staining of transient transgenic *S. psammophila* seedlings. (**B**) Transient transformation rate. Values are means ± SD; the difference between different letters is significant (*p* < 0.05), while the difference between the same letters is not significant (*p* > 0.05); *n* = 3.

To investigate the impact of secondary culture time on the transient transformation efficiency of *S. psammophila*, tissue culture seedlings of *S. psammophila* were infected with *Agrobacterium* GV3101 carrying the GUS gene at 20, 30, 45, and 60 d after secondary culture (OD_600_ value of 0.8, infection time of 3 h, co-culture duration of 48 h) (Figure 1). Although seedlings’ secondary culture for 20 d could be stained with GUS staining solution, the severe death and injury of seedlings after co-cultivation was not conducive to the statistical transformation rate and subsequent experiments. The *S. psammophila* of tissue culture seedlings at 30 d after secondary culture showed a deeper blue color, while the tissue culture seedlings at 45 and 60 d after secondary culture showed a slight blue color (Figure 1A), and the transient transformation efficiency was the highest after 30 d of secondary culture, with 53.33% (Figure 1B). In addition, from the cross-section of the stems and leaves of GUS-stained seedlings, GUS expression was found is in xylem cells and other cell types (Figure S1). Consequently, 30-day-old *S. psammophila* seedlings were identified as the optimal receptor material for subsequent transformation experiments.

### 2.2. Effect of Agrobacterium Concentration on Transient Transformation Efficiency of S. psammophila

**Figure 2:**
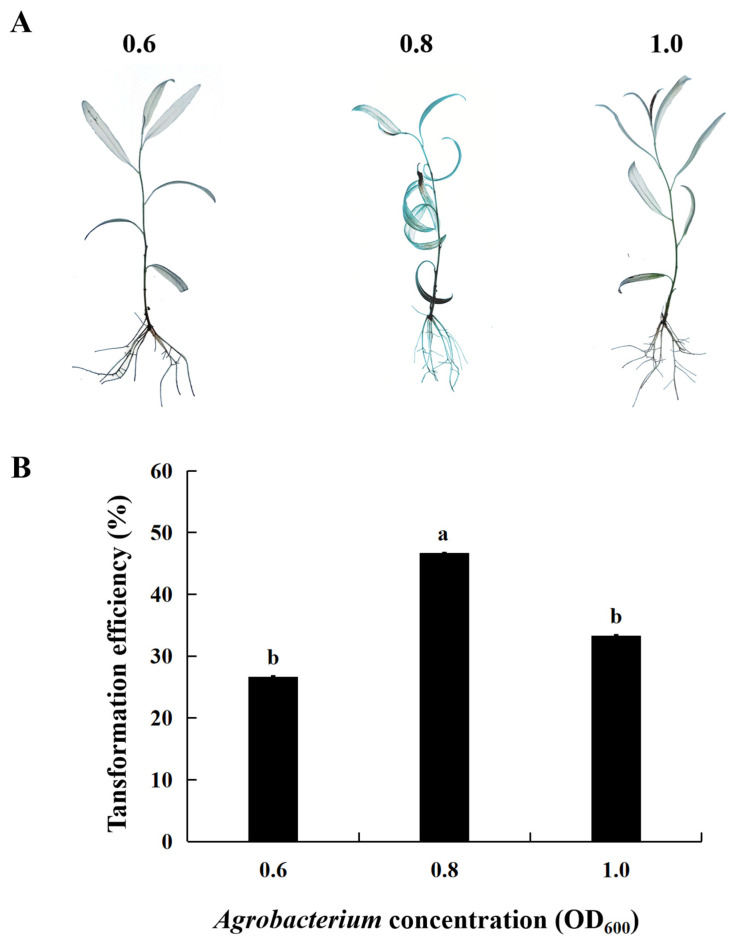
Effect of *Agrobacterium* concentration on transient transformation of *S. psammophila*. (**A**) GUS staining of transient transgenic *S. psammophila* seedlings. (**B**) Transient transformation rate. Values are means ± SD; the difference between different letters is significant (*p* < 0.05), while the difference between the same letters is not significant (*p* > 0.05); *n* = 3.

To evaluate the effect of *Agrobacterium* concentration on the transient transformation efficiency of *S. psammophila*, three concentrations of *Agrobacterium* were set in this experiment (OD_600_ = 0.6, 0.8, 1.0; secondary culture time of 30 d; infection time of 3 h; co-culture duration of 48 h). GUS staining results showed a light–dark–light change as the OD_600_ value varied from 0.6 to 1.0 (Figure 2A), with the transient transformation efficiency initially increasing and then decreasing (Figure 2B). Notably, at an OD_600_ value of 0.8, its transient transformation efficiency was 1.7 and 1.4 times greater than that observed at OD_600_ values of 0.6 and 1.0, respectively. Therefore, an *Agrobacterium* concentration of 0.8 was determined to be the most suitable for the transient transformation system of *S. psammophila* in this experiment.

### 2.3. Effect of Infection Time on Transient Transformation Efficiency of S. psammophila

**Figure 3:**
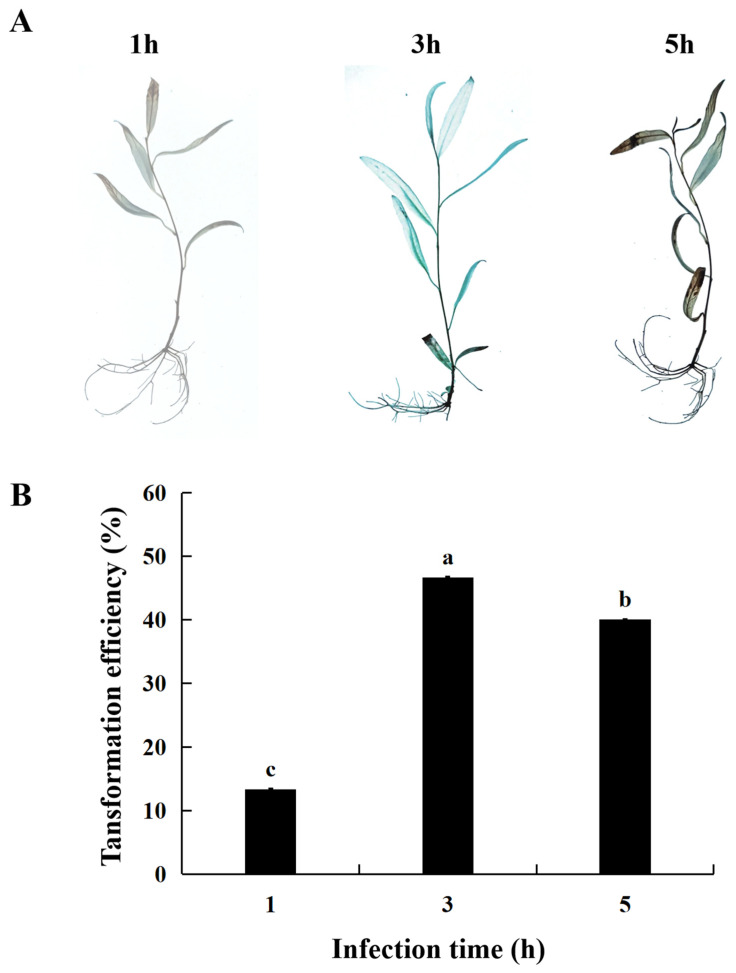
Effect of infection time on transient transformation of *S. psammophila*. (**A**) GUS staining of transient transgenic *S. psammophila* seedlings. (**B**) Transient transformation rate. Values are means ± SD; the difference between different letters is significant (*p* < 0.05), while the difference between the same letters is not significant (*p* > 0.05); *n* = 3.

The infection time of *Agrobacterium* is of paramount importance in transient transformation efficiency and also determines the efficiency of transient transformation. To investigate the effect of infection time, this experiment established three time points, 1, 3, and 5 h (secondary culture time of 30 d, OD_600_ value of 0.8, co-culture duration of 48 h) for infection transformation. When infected for 1 h, GUS staining was almost invisible. After being infected 3 or 5 h, the staining effect was darker than that observed in 1 h. However, when infected for 5 h, the tissue culture seedlings wilted severely and could not recover in the later stage (Figure 3A). And when the infection time was 3 h, the transient transformation efficiency reached 46.67% (Figure 3B). Therefore, in order to ensure the optimal condition of tissue culture seedlings for subsequent experiments, an infection time of 3 h was selected for the transient transformation system.

### 2.4. Effect of Co-Culture Duration on Transient Transformation Efficiency of S. psammophila

**Figure 4:**
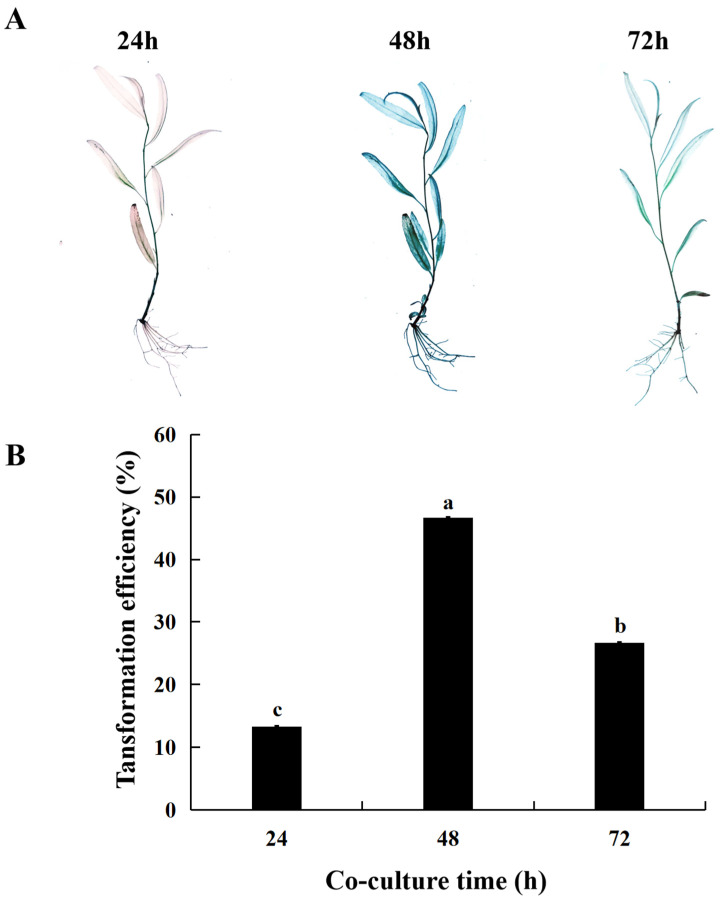
Effect of co-culture duration on transient transformation of *S. psammophila*. (**A**) GUS staining of transient transgenic *S. psammophila* seedlings. (**B**) Transient transformation rate. Values are means ± SD; the difference between different letters is significant (*p* < 0.05), while the difference between the same letters is not significant (*p* > 0.05); *n* = 3.

Co-cultivation can facilitate the invasion of *Agrobacterium* into transformed materials and improve transformation efficiency. However, if the co-culture duration is too long, it is easy to cause the excessive proliferation of *Agrobacterium*, affecting the survival of transformed materials. Therefore, too long a co-culture duration is not conducive to transformation. When the co-culture duration was 24 h, the transient transformation efficiency was the lowest, at 13.33%. When the co-culture duration extended to 48 h, the transient transformation rate achieved the maximum value of 46.67%. However, when co-cultured for 72 h, the conversion efficiency decreased, which was due to the absence of antibiotics in the co-culture medium, and the excessive proliferation of *Agrobacterium* resulted in the browning and softening of tissue culture seedlings as the duration of co-culturing increased, which was aligned with the low transformation efficiency observed (Figure 4). The results showed that co-culture for 48 h was the best co-culture duration for instantaneous transformation.

### 2.5. Verification of Transient Transformation System of S. psammophila

**Figure 5:**
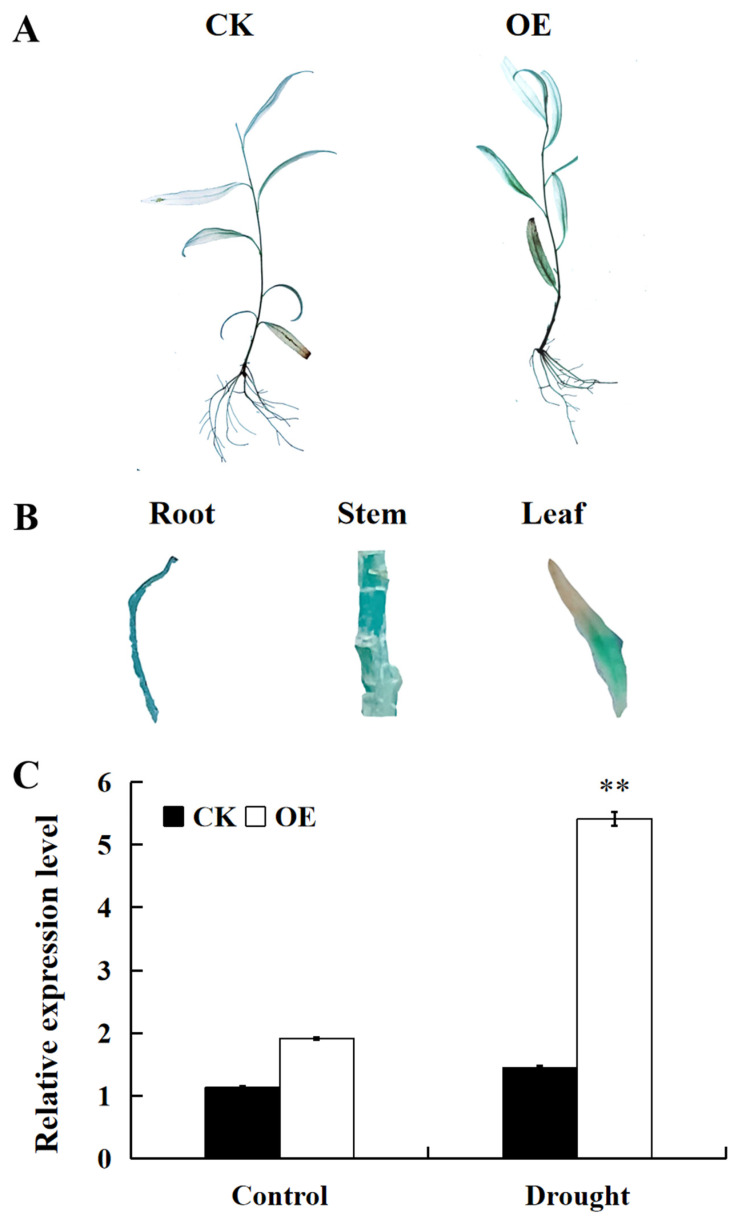
Identification of transient transgenic *S. psammophila* and expression analysis. (**A**) GUS staining of transient transgenic *S. psammophila* seedlings. (**B**) GUS staining of root, stem, and leaf of transient transgenic *S. psammophila* seedlings. (**C**) Transient expression analysis of *S. psammophila* stress resistance genes under abiotic stresses. CK: *S. psammophila* seedlings transformed with empty pCAMBIA1301; OE: *S. psammophila* seedlings overexpressing *SpPP2C80.* Values are means ± SD, ** indicates significant differences compared with the CK at the *p* < 0.01 level, *n* = 3.

To verify the feasibility of the transient transformation system, the function of the PP2Cs family gene *SpPP2C80* which was induced under drought stress was analyzed using this system [26]. The constructed plasmid overexpression of *SpPP2C80* was transiently transferred into *S. psammophila* tissue culture seedlings. The GUS staining results showed that both the transient transformation empty vector and *SpPP2C80* gene were stained blue in the seedlings (Figure 5A), and a blue color could be observed in its roots, stems, and leaves (Figure 5B). In addition, the expression of *SpPP2C80* in OE seedlings was higher than in the control, especially after drought treatment (Figure 5C).

**Figure 6:**
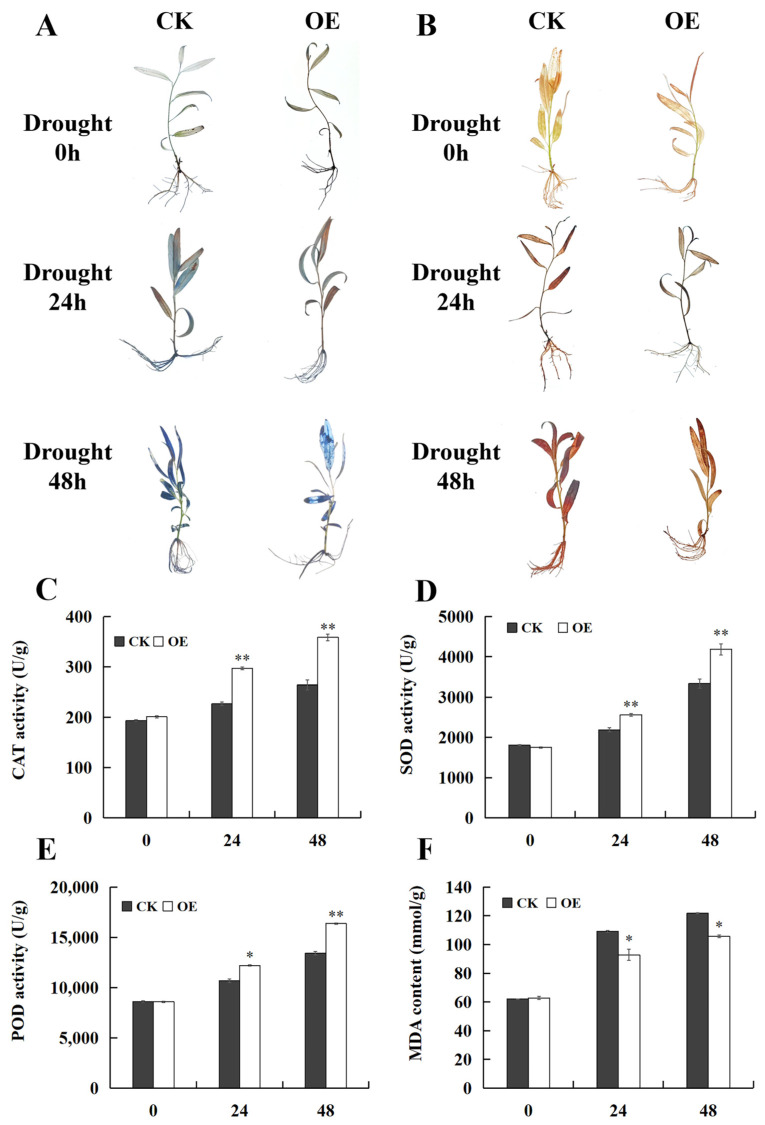
Physiological index analysis of *S. psammophila* seedlings of CK and OE under drought stress. (**A**) NBT and (**B**) DAB staining. (**C**) CAT activity levels. (**D**) SOD activity levels. (**E**) POD activity levels. (**F**) MDA content. CK: *S. psammophila* seedlings transformed with empty pCAMBIA1301; OE: *S. psammophila* seedlings overexpressing *SpPP2C80.* Values are means ± SD, * and ** indicate significant differences compared with the CK at the *p* < 0.05 level and *p* < 0.01 level, *n* = 3.

To further examine the resistance of transgenic seedlings with *SpPP2C80* to drought stress, seedlings were subjected to 200 mM D-mannitol for 0 h, 24 h, and 48 h for drought treatment. Drought stress causes the excessive production of reactive oxygen species (ROS) in plants, resulting in oxidative stress that damages plant cells and ultimately decreases plant growth and development. To further evaluate the effects of *SpPP2C80* overexpression on ROS accumulation, the levels of two primary ROS, H_2_O_2_ and O^2−^, were determined by performing 3, 3′-diaminobenzidine (DAB) and nitroblue tetrazolium (NBT) staining on OE and CK seedlings. DAB and NBT staining results between the OE and CK *S. psammophila* lines were not varied prior to drought stress. However, CK seedlings were darker than OE seedlings after drought treatment, with darker staining after 48 h than 24 h (Figure 6A,B). The analysis of antioxidant enzyme activities suggested that after 24 h of drought treatment, the catalase (CAT), superoxide dismutase (SOD), and peroxidase (POD) activities of OE seedlings were 1.30, 1.17, and 1.14 times higher than those of CK seedlings, respectively. However, after 48 h of drought treatment, the CAT, SOD, and POD activities of OE seedlings were 1.36, 1.26, and 1.22 times higher than those of CK seedlings, respectively (Figure 6C–E). In addition, the cell membrane permeability physiological indexes for malondialdehyde (MDA) of OE and CK seedlings was assessed, and the results suggested that OE seedlings had obviously lower values than CK seedlings under drought conditions (Figure 6F). The above results indicate that *SpPP2C80* could improve the drought resistance of transient transgenic *S. psammophila*.

**Figure 7:**
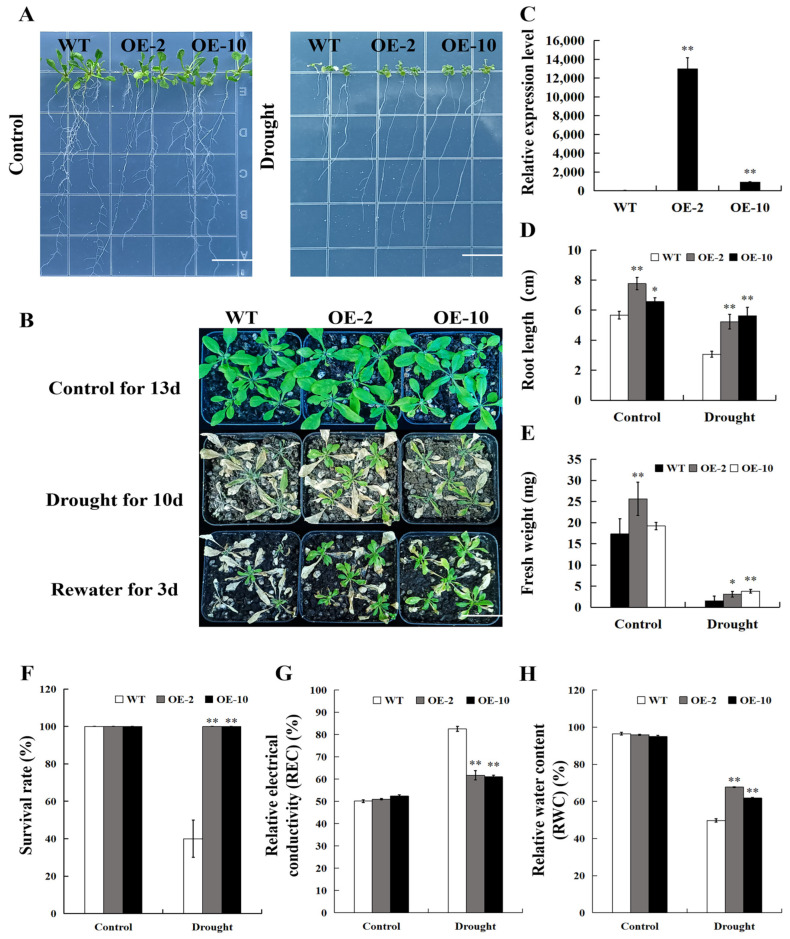
Analysis of drought tolerance of *Arabidopsis* under 1/2 MS medium and soil growth conditions. (**A**) Photos of WT and transgenic *Arabidopsis* seedlings under normal and drought conditions. (**B**) Photos of WT and transgenic seedlings under normal conditions and drought treatment. WT and two independent transgenic lines grew in soil, with 5 seedlings per genotype. Soil treatment includes three biological replicates. (**C**) Relative expression levels of *SpPP2C80* in WT and transgenic *Arabidopsis*. (**D**) Root length. (**E**) Fresh weigh. (**F**) Survival rate. (**G**) Relative electrical conductivity (REC). (**H**) Relative water content (RWC) of WT and transgenic *Arabidopsis* under normal and drought conditions. Bars = 5 cm. Values are means ± SD, * and ** indicate significant differences compared with the CK at the *p* < 0.05 level and *p* < 0.01 level, *n* = 3.

To verify the accuracy of this result, 2 T3 homozygous lines (OE-2 and OE-10) with high *SpPP2C80* expression levels were selected from over 30 transgenic *Arabidopsis* lines for drought experiment analysis (Figure 7C). Firstly, the wild-type (WT) and two transgenic lines were exposed to plate drought stress treatment. The results showed that under normal conditions, the root length and fresh weight of transgenic *Arabidopsis* grown in 1/2 Murashige and Skoog (MS) medium were greater than those in WT (Figure 7D–E), indicating that the overexpression of *SpPP2C80* promoted *Arabidopsis* growth to some extent, but this supposition needs further experimental verification. However, OE-2 and OE-10 exhibited better growth conditions in the medium containing D-mannitol (Figure 7A), with root lengths 1.53 and 1.61 times than that of WT (Figure 7D), and fresh weights 1.31 and 1.39 times that of WT (Figure 7E), respectively. Secondly, seedlings that grew on normal medium for 10 d were transplanted into soil for one month, and then, drought treatment was performed. After stopping watering for 10 d, WT leaves wilted more severely and could not recover after resuming watering, while OE-2 and OE-10 regained vitality after resuming watering (Figure 7B). The survival rate of OE-2 and OE-10 was 100%, while WT was only 40% (Figure 7F). Leaf relative electrical conductivity (REC) and relative water content (RWC) are vital biochemical markers that reflect plant drought tolerance. The REC increased, while RWC reduced, when plants faced drought stress. As expected, OE-2 and OE-10 had lower REC (Figure 7G) and higher RWC (Figure 7H) values under drought conditions, compared with the WT.

**Figure 8:**
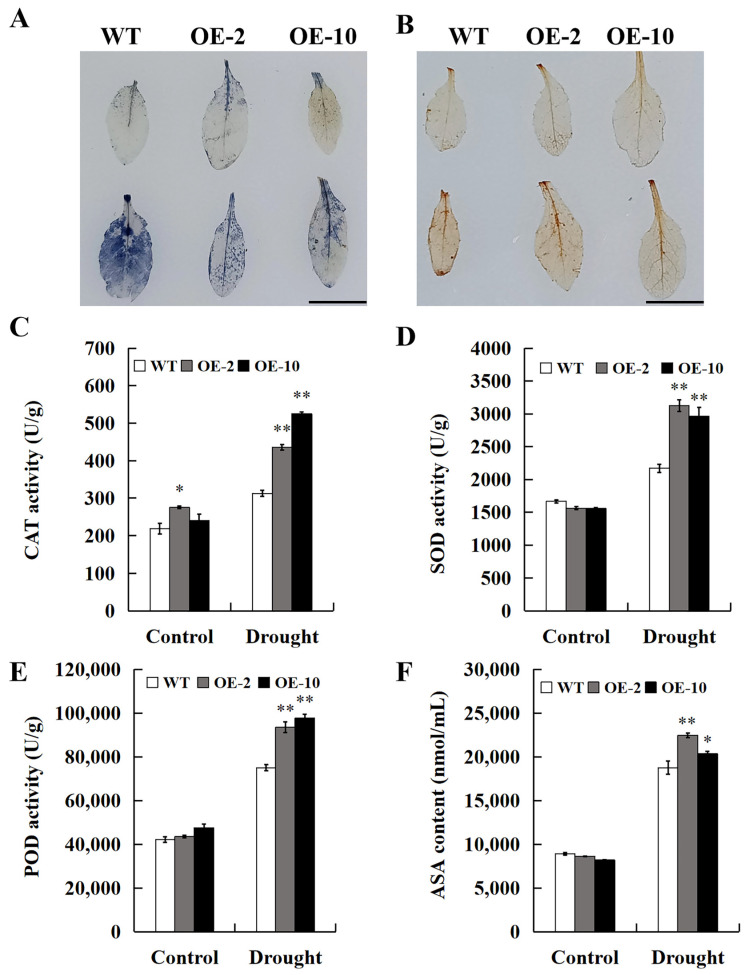
Study on antioxidant enzyme activity in WT and transgenic *Arabidopsis*. (**A**) NBT and (**B**) DAB staining of rosette leaves from drought-treated and normally grown WT and transgenic *Arabidopsis* plants. (**C**–E) Analysis of CAT, SOD, and POD activities under control and drought conditions. (**F**) AsA content. Bars = 5 cm. Values are means ± SD, * and ** indicate significant differences compared with the WT at the *p* < 0.05 level and *p* < 0.01 level, *n* = 3.

Subsequently, WT and transgenic *Arabidopsis* grown in water-deficient conditions for 10 d were analyzed by DAB and NBT staining. Under water scarcity conditions, the leaves of WT *Arabidopsis* exhibited more pronounced and widespread staining than transgenic *Arabidopsi*s (Figure 8A,B). On the other hand, the plant antioxidant defense system removes ROS to avoid the damage induced by oxidative stress, and CAT, SOD, and POD play vital roles in detoxifying ROS overproduction in plants. The enzyme activities analysis results indicate that the CAT, SOD, and POD activities of transgenic *Arabidopsis* were 1.53-fold, 1.39-fold, and 1.27-fold higher than those of WT *Arabidopsis*, respectively (Figure 8C–E). In addition, ascorbic acid (AsA) is an important antioxidant involved in ROS scavenging in plants, so the AsA level was calculated. The results indicate that the AsA content of transgenic *Arabidopsis* was prominently greater than that of WT *Arabidopsis* (Figure 8F). These results indicate that the overexpression of *SpPP2C80* enhances the drought resistance of transgenic *Arabidopsis*, which is consistent with the transient transformation of *SpPP2C80* by *S. psammophila*.

The above findings revealed that the transient transformation system of *S. psammophila* was successfully utilized to transfer genes into the seedlings of *S. psammophila,* and this transformation system could serve as a tool to investigate the functions in *S. psammophila*.

## 3. Discussion

This study investigated the effects of secondary culture time, *Agrobacterium* concentration, infection time, and co-culture duration on the transient transformation efficiency of *S. psammophila* tissue culture seedlings. The effect of plant cotyledons on *Agrobacterium* varies with different cultivation times. Young plant tissue materials as transformation materials have poor cell resistance and struggle to withstand the toxicity of *Agrobacterium*, which can reduce the transformation rate of plasmids. In contrast, plant tissue with longer cultivation times has thicker cuticles, making it difficult for *Agrobacterium* to penetrate plant cells [17]. Therefore, screening the optimal cultivation time of *S. psammophila* tissue culture seedlings is crucial for establishing an efficient *S. psammophila* transient transformation system. Hou et al. chose to cultivate 35-day-old *Tamarix chinensis* tissue cultured seedlings as the material for transient transformation [35]. Guan et al. believe that the most suitable germination time for the instant transformation of *Paeonia lactiflora* tissue culture seedlings is 30 d [17]. This study found that the transformation efficiency of *S. psammophila* tissue cultured seedlings was higher after 30 d of secondary culture, with GUS staining exhibiting deeper and more extensive staining.

Research has shown that the concentration of *Agrobacterium* directly determines the instantaneous conversion efficiency of plants [13]. An excessive concentration of *Agrobacterium* can cause damage to plants, while an insufficient concentration cannot provide sufficient *Agrobacterium* [36]. During the construction of the transient transformation system for WT *Syntrichia caninervis*, it was found that the GUS expression was highest and the blue block was deepest when the OD_600_ value of *Agrobacterium* concentration was cultured to 0.8, which was most suitable for the transient transformation of WT *S. caninervis* [37]. For the transient transformation of Xinjiang apples, the OD_600_ of 0.6 was chosen for the highest conversion efficiency [22]. In this study, the optimal transient transformation efficiency of *S. psammophila* was achieved when the OD_600_ value of *Agrobacterium* was 0.8.

The infection of *Agrobacterium* requires a certain amount of time, ensuring sufficient *Agrobacterium* infiltration into plant cell tissues and preventing damage to the plant. In previous research on establishing transient transformation systems for *Malus sieversii* [38], *Pinus tabulaeformis* [39], and *Iris foetidissima* [20], bacterial infection time was screened. The results indicate that a too-short infection time can render the infection ineffective, while a too-long infection time can lead to plant damage. In our study, the optimal infection time for the *Agrobacterium-*mediated transient transformation of *S. psammophila* was 3 h.

*Agrobacterium* requires a certain length of co-culture duration to achieve maximum transformation. In order to promote the proliferation of *Agrobacterium* that has infiltrated plant cell tissues and ensure the entry of T-DNA into plant cells for transient expression, different co-culture durations affect the expression level of GUS genes. An insufficient co-culture duration prevents the transformation of *Agrobacterium*, but too long a co-culture duration can cause the inordinate growth of *Agrobacterium* and damage plants. The best co-culture duration of *I. foetidissima* is 4 d [20]. However, the most suitable co-culture durations for *Rhododendron yunnanense* and *Cunninghamia lanceolata* are 5 d [40,41]. In this study, 48 h was the optimum co-culture duration for the transient transformation in *S. psammophila;* this result is the same as the time required for the genetic transformation co-culture of *Populus angustifolia* and *Populus balsamifera* [42].

The transient expression of plants has been widely used in gene function verification. For plants with no mature genetic transformation system, it is very difficult to study the gene function associated with an obvious phenotype. Therefore, it is necessary to establish a transient transformation system for gene function exploration. Currently, transient transformation systems have been widely used to study gene functions in plants [5]. *ThZFP1* was overexpressed in *Tamarix hispida* through an *Agrobacterium-*mediated transient transformation system, which enhanced its tolerance to salt and osmotic stress by accumulating proline and increasing SOD and POD activities [43]. Moreover, the *Agrobacterium*-mediated transient transformation system can also be applied to promoters, transcription factors, protein subcellular localization, and interaction analysis. In *Poncirus trifoliata,* utilizing the transient transformation system established earlier, it was found that PERFI09 can directly bind to the *PtrPrx1* promoter, positively activate its expression, effectively eliminate reactive oxygen species, and thus improve plant cold tolerance [44]. *Vitis vinifera* locates flavonoid biosynthetic enzymes in the cytoplasm and nucleus of *V. vinifera* through a transient transformation system established using protoplasts as receptor materials [45]. However, transient transformation systems also have certain limitations, as they can only be used for short-term genetic research and lack stability.

Drought is one of the main abiotic constraints that threaten plant growth and crop production [46]. When the available water in the environment is lower than what plants need for normal growth, this can lead to a decrease in water absorption and stomatal closure and a decrease in photosynthesis rate, ultimately affecting the normal growth of plants and crop yield [47,48]. PP2C protein phosphatases participate in plant growth and cell differentiation and play a significant role in the adaptation and signal transduction of the drought response [49,50]. *TaPP2C-a10* negatively regulates drought resistance in *Arabidopsis* [51]. *CePP2C19* overexpression improved the growth status of *Arabidopsis* under drought stress and enhanced its ability to respond to drought, while silencing significantly reduced drought resistance [52]. In this study, *SpPP2C80* overexpression increased the viability and RWC of *Arabidopsis*, reduced REC, and maintained good growth under drought conditions (Figure 7). As vital protective enzymes in plant cells, antioxidant enzymes, whose activity raises dramatically under drought condition, scavenge excessive ROS in plants to sustain cellular homeostasis [53,54]. The overexpression of *BpPP2C1* in *B. platyphylla* resulted in higher SOD, POD, and CAT activities under salt stress [14]. Compared with WT *Arabidopsis*, the overexpression of *ZmPP2C15* resulted in higher SOD, POD, and CAT activities but lower MDA content in *Arabidopsis* in drought stress [55]. In addition, AsA is a major antioxidant, which ensures the protection of plant cells against ROS generated both during normal physiological processes as well as by biotic and abiotic stresses [56]. AsA oxidation leads to monodehydroascorbate which can be recycled back to AsA because of MDHAR using NAD(P)H as the reductant [57]. In our study, under drought conditions, compared with CK, *S. psammophila* seedlings with the transient transformation of the *SpPP2C80* gene showed higher SOD, POD, and CAT activities and lower MDA content, and *SpPP2C80* overexpression in *Arabidopsis* also had similar results (Figure 6 and Figure 8). These results indicated that *SpPP2C80* overexpression improved the capacity of ROS scavenging, kept much better cell membrane stability, and improved the drought tolerance of the plant. Furthermore, the same results as the functional study of *SpPP2C80* were obtained through transient transformation and stable inheritance, which demonstrated the high efficiency and usability of transient transformation.

## 4. Materials and Methods

### 4.1. Plant Materials and Growth Conditions

We washed the stem segments of *S. psammophila* with tap water for 2 h, sterilized them with 10% NaClO_2_ for 15–20 min in a super clean workbench, sterilized them twice with 75% alcohol for 5–10 min each time, and finally rinsed them 4–5 times with sterile water. We removed them and used sterile filter paper to absorb surface moisture and inserted them into the starting 1/2 MS medium (1/2 MS + 20 g/L sucrose + 6.5 g/L agar, pH = 5.9) for 30 d of cultivation, placed under the conditions of a 16/8 h (light/dark) photoperiod at 25 ± 2 ℃. We cut off about 1 cm of newly grown tender branches from *S. psammophila* stem segments and inserted them into new 1/2 MS medium (1/2 MS + 0.5 mg/L 6-Benzylaminopurine (6-BA) + 0.05 mg/L Naphthalene acetic acid (NAA) + 20 g/L sucrose + 6.5 g/L agar, pH = 5.9) for secondary culture. We selected tissue culture seedlings that had been secondary cultured for 20 d, 30 d, 45 d, and 60 d as instant transformation materials.

*Arabidopsis* seeds were surface-sterilized and seeded on 1/2 MS medium. After growing for 10 d, they were transplanted into perlite and soil (1:3 *v/v*) and grown for one month before being used for experiments. Ultimately, germinated seeds and seedlings were cultivated at standard conditions (22 °C, 16/8 h (light/dark)).

### 4.2. Plasmid Construction and Agrobacterium Culture

The pCAMBIA1301 empty vectors were introduced into *Agrobacterium* GV3101 by the freeze–thaw method. *Agrobacterium* cells were spread in selective LB solid medium plus 50 mg/L kanamycin (Kan) and 50 mg/L rifampin (Rif) and then cultivated at 28 °C for 48 h–72 h. The single colony was picked from LB plates and inoculated in 1 mL of LB liquid medium that contained antibiotics and was incubated for 24 h at 28 °C with shaking (220  rpm). The turbid bacterial solution was diluted to new LB liquid medium (adding 50 mg/L Kan and 50 mg/L Rif) at 1:50 and overnight cultivation. Then, the bacteria were collected by centrifugation at 5000 rpm for 15 min and resuspended with infecting solution (1/2 MS + 1 M D-mannitol + 10 mM MgCl_2_ +10 mM CaCl_2_ +10 mM 2-Morpholinoethanesulphonic acid (MES) +200 µM Acetosyringone (AS) +1.5 mg/L Kinetin (KT) + 5 mg/L 2, 4-Dichlorophenoxyacetic acid (2, 4-D) + 0.02% (*v/v*) Tween 20) for *S. psammophila*, and the OD_600_ value of the infection solution was adjusted to the required concentration for conversion, and then, they were left at room temperature for 1 h for conversion.

### 4.3. Agrobacterium-Mediated Transient Transformation and Screening of Transient Transformation Conditions

The transient transformation of tissue culture seedlings of *S. psammophila* mediated by *Agrobacterium* was carried out according to the following steps: (1) Soak the tissue culture seedlings of *S. psammophila* in high-permeability treatment solution (1 M D-mannitol) for 5–10 min. (2) Place the soaked tissue cultured seedlings of *S. psammophila* into an infection solution containing *Agrobacterium*. (3) Vacuumize for 5–10 min; then, transfer them to a constant-temperature shaking incubator at 24–28 °C and 120–130 rpm for infection for 1, 3, or 5 h. (4) Wash the infected tissue culture seedlings of *S. psammophila* with sterile water 3–5 times, absorb the water with sterile filter paper, and insert them into solid co-culture medium for dark cultivation for 24, 48, or 72 h.

**Figure 9:**
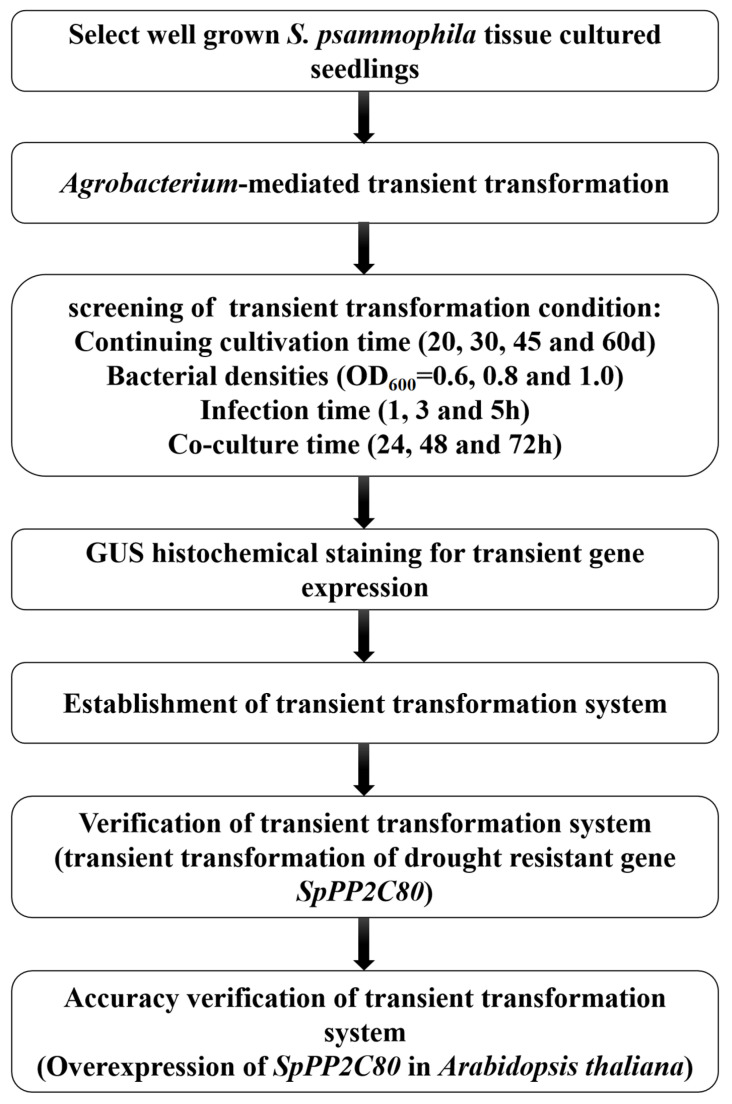
Flow chart of *Agrobacterium*-mediated transient transformation system for the development of transgenic *S. psammophila* plants.

We selected the conditions for the *Agrobacterium*-mediated transient transformation of *S. psammophila* tissue culture seedlings, including secondary culture time (20, 30, 45, and 60 d), *Agrobacterium* concentration (0.6, 0.8, and 1.0 OD_600_), infection time (1, 3, and 5 h), and co-culture duration (24, 48, and 72 h). The experimental design is shown in Table 1. This experiment was repeated 3 times, and 5 seedlings were transformed each time. The summary of the experimental process is shown in Figure 9.

### 4.4. GUS Staining Assays

The transgenic tissue culture seedlings of *S. psammophila* were soaked in GUS staining solution (0.52 g/L X-Glvc + 200 mM NaH_2_ + 200mM Na_2_H + 100 mM K_3_[Fe(CN)_6_] + 100 mM K_4_[Fe(CN)_6_]) overnight in the dark at 37 °C. The stained samples were repeatedly decolorized with 75% ethanol until chlorophyll was no longer visible. A blue color in tissue was considered as an indicator of positive transgenic explants. Transformation efficiency was computed according to the following equation: transformation efficiency (%) = (number of seedlings stained/total number of seedlings) × 100. Each group of experiments were repeated three times.

### 4.5. Transient Overexpression of SpPP2C80 in S. Psammophila and Drought Resistance Analysis

The CDS of *SpPP2C80* was cloned into a pCAMBIA1301 plant expression vector with CaMV 35S and transformed into DH5α; positive recombinants were identified by sequencing. Then, the positive recombinant plasmid (pCAMBIA1301-*SpPP2C80*) was transformed into GV3101 and cultivated according to method 5.2. Then, the steps of method 5.3 were used to transiently transform *S. psammophila* to obtain transiently transgenic *S. psammophila* tissue culture seedlings.

Then, 30-day-old seedlings of the transient transgenic *S. psammophila* seedlings (OE and CK) subjected to 200 mM D-mannitol for 0 h, 24 h, and 48 h for drought treatment were harvested.

### 4.6. Quantitative Real-Time PCR (qRT-PCR)

Total RNA was isolated from transient transformed *S. psammophila* tissue culture seedlings (normal and drought) and transgenic *Arabidopsis* leaves using the FastPure Universal Plant Total RNA Isolation Kit (Nanjing Vazyme Biotech Co., Lid., Nanjing, China), and one microgram of total RNA was reverse-transcribed using SuperScript III (+gDNA wiper) as described in the manufacturer’s protocol. qRT-PCR was performed using Taq Pro Universal SYBR qPCR Master Mix (Nanjing Vazyme Biotech Co., Lid, Nanjing, China). Three replicate biological experiments were performed. Relative expression levels were calculated using the formula 2^−∆∆C(T)^, and Ubiquitin-conjugating enzyme E2 (UBC) [1] and *AtActin* [58] were chosen as reference genes for *S. psammophila* drought treatments and *Arabidopsis*, respectively. All the primer sequences are listed in Table S1.

### 4.7. Ectopic Expression of SpPP2C80 in Arabidopsis and Drought Resistance Analysis

*Agrobacterium* containing pCAMBIA1301-*SpPP2C80* was transferred into *Arabidopsis* by the flower invasion method to obtain transgenic *Arabidopsis* seeds [59].

More than 30 transgenic *Arabidopsis* lines were obtained in the experiment, and 2 T3 homozygous lines that exhibited relatively high transcriptional levels of *SpPP2C80* in transgenic *Arabidopsis* were selected for further analysis. Then, 10-day-old seedlings were transplanted to 1/2 MS medium plus 200 mM D-mannitol (simulated drought experiment) and a mixture of perlite and soil (1:3 *v/v*). After 4 weeks of normal growth in soil, watering was stopped for 10 d, and then, watering was restored for 3 d.

### 4.8. Physiological Analysis

*Arabidopsis* leaves (normal and drought) and transient transgenic *S. psammophila* seedlings (OE and CK) were stained using DAB and NBT. And the leaf REC, RWC, and enzyme activity were measured. REC and RWC were measured as described previously [34]. CAT, POD, SOD, AsA, and MDA were measured using the Beijing Solaibao Technology Co., Ltd. reagent kit.

### 4.9. Statistic Analysis

SPSS 26.0 software was used for the statistical analysis of all data, and significant differences between the samples were determined using Student’s *t* test. Multiple groups of data were compared using ANOVA with an LSD multiple comparisons test.

## 5. Conclusions

The present study established an efficient *Agrobacterium*-mediated transient transformation system for *S. psammophila*. This system enabled the transient overexpression of resistance genes in *S. psammophila* within a short time frame and allowed for the assessment of the stress resistance in the transgenic plants. The experimental results demonstrated that *SpPP2C80* enhances the resistance of *S. psammophila* to drought stress. Moreover, the effectiveness of the established transient transformation system was confirmed by the overexpression of *SpPP2C80* in *Arabidopsis.* Consequently, this system will facilitate the functional investigation of genes in *S. psammophila*.

## Figures and Tables

**Table 1:** Experimental design of transient transformation factors for *S. psammophila* tissue culture seedlings.

Secondary Culture Time (d)	*Agrobacterium* Concentration (OD600)	Infection Time (h)	Co-Culture Duration (h)
20, 30, 45, 60	0.8	3	48
30	0.6, 0.8, 1.0	3	48
30	0.8	1, 3, 5	48
30	0.8	3	24, 48, 72

## Data Availability

Data generated in the current work are provided in the manuscript.

## Supplementary Materials

The following supporting information can be downloaded at: https://www.mdpi.com/article/10.3390/ijms252312934/s1.
