# Supplementary figures and images for: Establishment and Validation of an Efficient Agrobacterium Tumefaciens-Mediated Transient Transformation System for Salix Psammophila
Source: Int J Mol Sci. 2024 Dec 1;25(23):12934. doi: 10.3390/ijms252312934 (PMC11641538; doi:10.3390/ijms252312934)

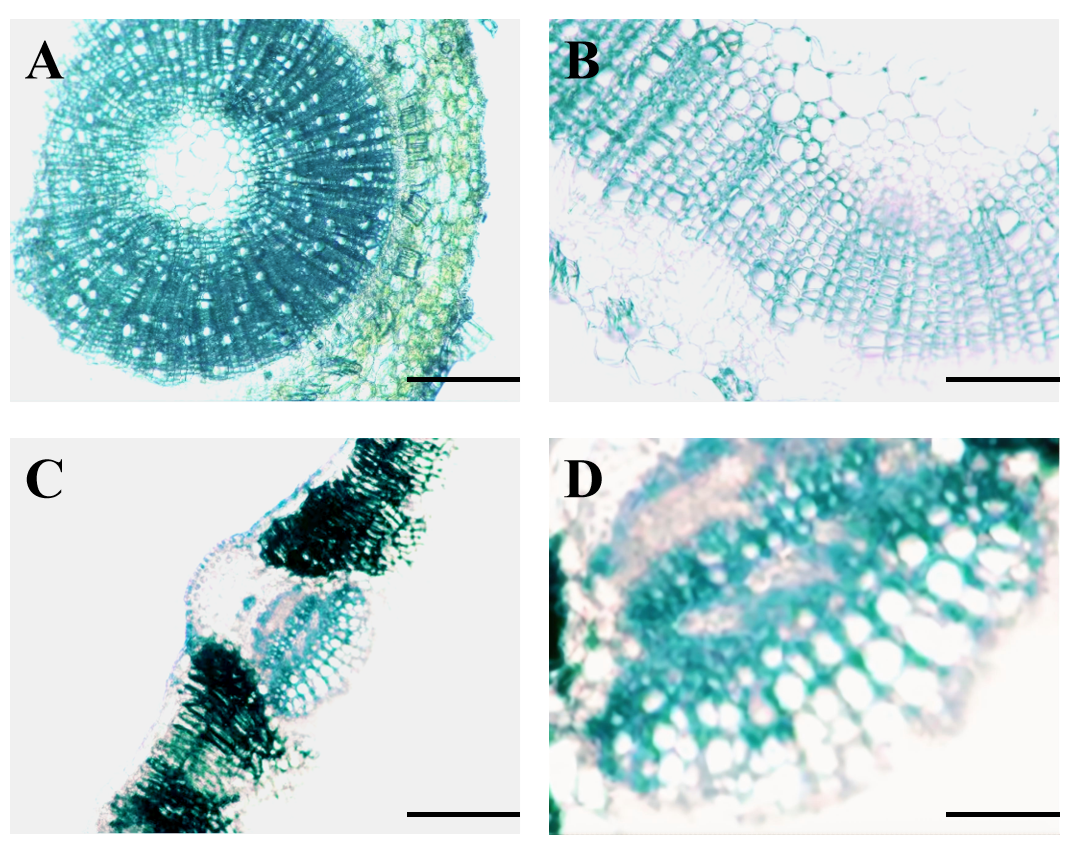

Supplement: Supplementary file 1 [file ijms-25-12934-s001.zip › Figure S1.tif]

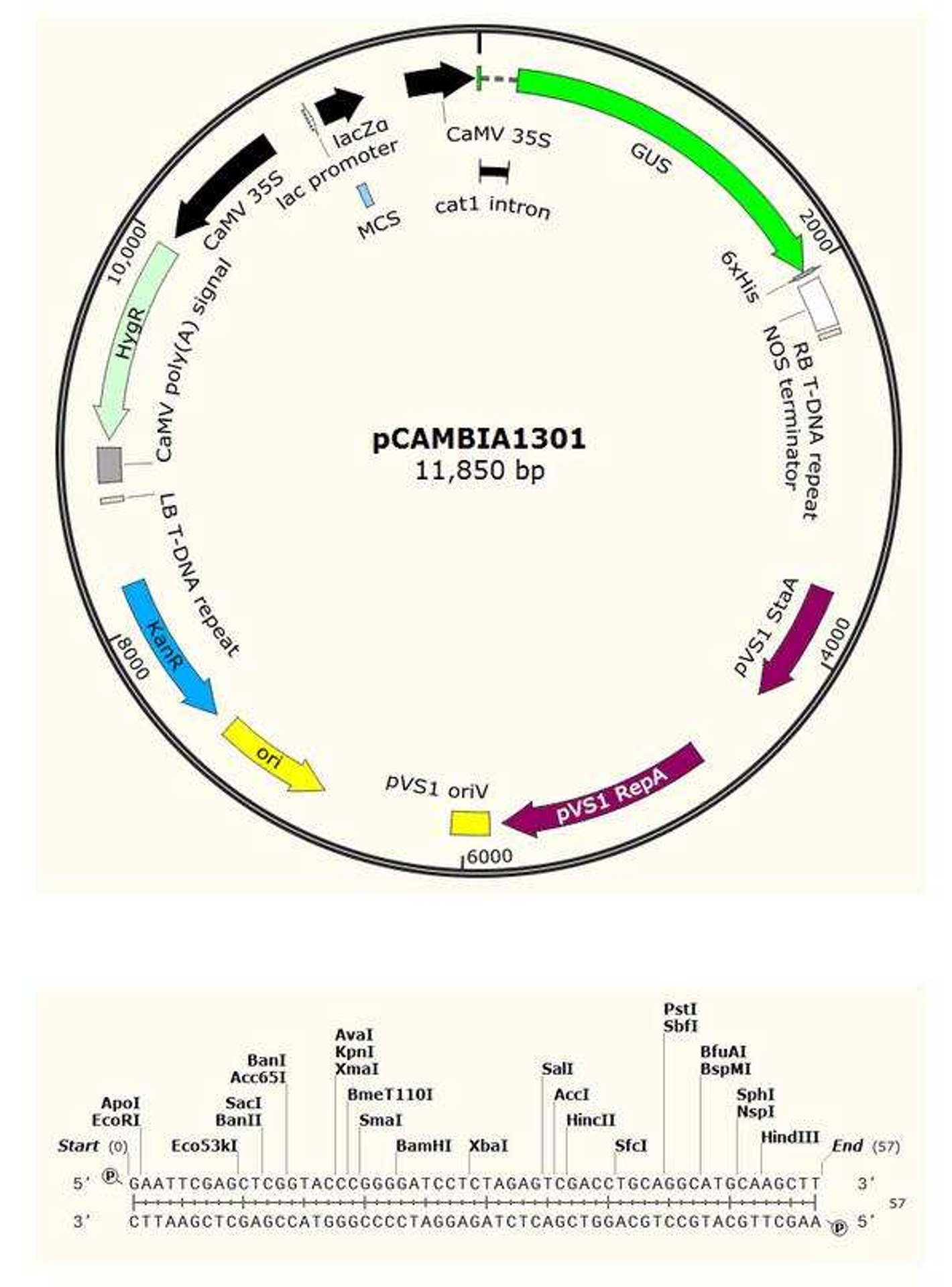

Supplement: Supplementary file 1 [file ijms-25-12934-s001.zip › Figure S2.tif]
